# The deubiquitylating enzyme UCHL3 regulates Ku80 retention at sites of DNA damage

**DOI:** 10.1038/s41598-018-36235-0

**Published:** 2018-12-17

**Authors:** Ryotaro Nishi, Paul W. G. Wijnhoven, Yusuke Kimura, Misaki Matsui, Rebecca Konietzny, Qian Wu, Keisuke Nakamura, Tom L. Blundell, Benedikt M. Kessler

**Affiliations:** 1 The Wellcome Trust/Cancer Research UK Gurdon Institute and Department of Biology, University of Cambridge, Tennis Court Road, Cambridge, CB2 1QN United Kingdom; 2 Department of Biomedical Sciences, College of Life Sciences, Ritsumeikan University, Shiga, 525-8577 Japan; 3 Target Discovery Institute, Nuffield Department of Medicine, University of Oxford, Oxford, OX3 7FZ United Kingdom; 4 Department of Biochemistry, University of Cambridge, Sanger Building, Tennis Court Road, Cambridge, CB2 1GA United Kingdom; 5 Present Address: Bioscience Oncology, IMED Biotech Unit, AstraZeneca, Cambridge, United Kingdom

## Abstract

Non-homologous end-joining (NHEJ), which can promote genomic instability when dysfunctional, is a major DNA double-strand break (DSB) repair pathway. Although ubiquitylation of the core NHEJ factor, Ku (Ku70-Ku80), which senses broken DNA ends, is important for its removal from sites of damage upon completion of NHEJ, the mechanism regulating Ku ubiquitylation remains elusive. We provide evidence showing that the ubiquitin carboxyl-terminal hydrolase L3 (UCHL3) interacts with and directly deubiquitylates one of the Ku heterodimer subunits, Ku80. Additionally, depleting UCHL3 resulted in reduced Ku80 foci formation, Ku80 binding to chromatin after DSB induction, moderately sensitized cells to ionizing radiation and decreased NHEJ efficiencies. Mechanistically, we show that DNA damage induces UCHL3 phosphorylation, which is dependent on ATM, downstream NHEJ factors and UCHL3 catalytic activity. Furthermore, this phosphorylation destabilizes UCHL3, despite having no effect on its catalytic activity. Collectively, these data suggest that UCHL3 facilitates cellular viability after DSB induction by antagonizing Ku80 ubiquitylation to enhance Ku80 retention at sites of damage.

## Introduction

Our genomes are constantly threatened by both endogenous and exogenous sources of genotoxic stress. If the resulting DNA lesions are left unrepaired or are repaired improperly, this can lead to cellular dysfunction, cell senescence, cell death or tumorigenesis^1^. DNA double-strand breaks (DSBs), which can be caused for example by ionizing radiation (IR), DNA replication fork collapse and certain types of anti-cancer medicines, are perhaps the most harmful DNA lesions and are mainly repaired either by classical non-homologous end-joining (c-NHEJ) or by homologous recombination (HR). In mammalian cells, HR is initiated by a process referred to as DNA-end resection via the actions of the MRE11-RAD50-NBS1 (MRN) complex and CtIP, resulting in the formation of 3′ single-stranded DNA (ssDNA) overhangs that can be further extended by exonucleases such as EXO1, DNA2 and EXD2^2–5^. Following strand invasion into an undamaged sister chromatid with the aid of factors including RPA, RAD51, BRCA1, BRCA2, PALB2, USP11 and chromatin remodeling complexes, the DNA sequence is copied from the sister chromatid by DNA replication^6–9^. Consequently, HR is generally a faithful DSB repair pathway when restricted to S and G2 phases of the cell cycle. In contrast to HR, c-NHEJ functions throughout the cell cycle except during mitosis and is responsible for most IR-induced DSB repair even in S and G2 phases^10,11^. C-NHEJ is initiated when broken DNA ends are sensed by Ku, a heterodimer composed of Ku70/XRCC6 and Ku80/XRCC5. Ku in turn promotes recruitment of DNA-dependent protein kinase catalytic subunit (DNA-PKcs) to form the DNA-PK holoenzyme^12^. Subsequently, additional c-NHEJ factors, most notably XRCC4, DNA Ligase IV (LIG4), XLF and PAXX are recruited to sites of damage to promote ligation of the two broken ends directly, or after DNA-end processing by the nuclease Artemis, specialized DNA polymerases and other accessory factors such as polynucleotide kinase-phosphatase (APLF)^10,13–16^. Significantly, biochemical and structural studies have shown that Ku forms a basket-shaped structure with a DNA double helix able to pass through its central channel, suggesting that Ku must become topologically trapped on DNA after completion of c-NHEJ^17^. As such sterically trapped Ku presumably interferes with subsequent DNA replication and transcription, Ku removal after c-NHEJ is likely to be crucial for maintaining genome integrity^18,19^. Thus far, it has been revealed that poly-ubiquitylation of Ku mediated by Lys48-linked ubiquitin chains is important for Ku removal from chromatin in human cells, and from closed double-stranded DNA (dsDNA) in *Xenopus laevis* egg extracts^20–24^. In addition to removing Ku from chromatin upon completion of c-NHEJ, there are several reports implying that Ku ubiquitylation regulates DSB repair pathway choice by employing different E3 ligases (RNF8 or RNF138) in a cell cycle-coupled manner^20,22^. Furthermore, inhibition of Ku ubiquitylation by depleting various ubiquitylation enzymes, results in increased cellular sensitivity to IR, further supporting the physiological importance of Ku ubiquitylation. While ubiquitylation is regulated by ubiquitylating enzymes comprising E1 activating enzymes, E2 conjugating enzymes and E3 ligases to promote covalent attachment of ubiquitin to a given substrate, it is evident that the ubiquitylation status of the substrate is also strongly affected by the reverse reaction—deubiquitylation—which is carried out by deubiquitylating enzymes (DUBs)^25,26^. While several E3 ligases have been implicated in Ku ubiquitylation, no DUBs antagonizing Ku ubiquitylation have yet been identified. Here, we show that the DUB UCHL3 is recruited to DNA damage sites, and interacts with and deubiquitylates Ku80 to promote c-NHEJ. In addition, siRNA-mediated depletion of UCHL3 causes a DNA repair defect that is largely restored upon complementation of cells with wild-type UCHL3, which correlates with depletion or genetic deletion of *UCHL3* moderately sensitising cells to IR. Mechanistically, we show that UCHL3 depletion results in reduced Ku80 foci formation upon IR, and chromatin retention upon phleomycin treatment in a manner that is reversed by wild-type UCHL3 expression. Lastly, we provide evidence showing that DNA damage-induced UCHL3 phosphorylation (which is dependent on UCHL3 catalytic activity, ATM kinase and downstream NHEJ factors) promotes destabilization of UCHL3, suggesting that UCHL3 may dissociate from Ku80 to allow Ku ubiquitylation and subsequent removal from DNA.

## Results

### UCHL3 interacts with Ku80 and is recruited to DNA damage sites in a manner dependent on its catalytic activity

**Figure 1:**
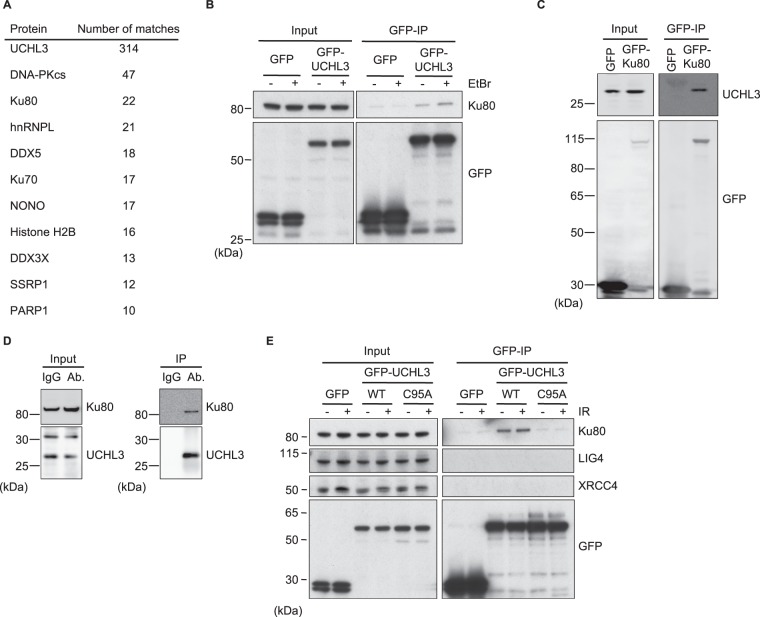
UCHL3 interacts with Ku80 and is recruited to sites of damage in a catalytic activity dependent manner. **(A)** Summary of mass spectrometry analysis of immunoprecipitate with an anti-GFP antibody from GFP-UCHL3 expressing cells. The hits obtained from GFP-UCHL3 over-expressing cells, but not detected with GFP-expressing cells (control) are shown with the number of peptide matches. Only proteins related to DNA damage responses are listed. **(B**,**C)** U2OS cells were transfected with a plasmid expressing GFP-UCHL3 **(B)** or GFP-Ku80 **(C)**. The soluble fraction was subjected to immunoprecipitation with an anti-GFP antibody with or without EtBr followed by immunoblotting with the indicated antibodies. Transfection with the plasmid expressing GFP was used as a negative control. **(D)** The soluble fraction of U2OS cells was subjected to immunoprecipitation with an anti-UCHL3 antibody (Ab) or rabbit IgG (IgG) followed by immunoblotting with the indicated antibodies. **(E)** U2OS cells were transfected with a plasmid expressing GFP-UCHL3 (wild-type: WT) or catalytically inactive mutant (C95A) and then treated with 10 Gy IR or mock treated. The soluble fraction was subjected to immunoprecipitation with an anti-GFP antibody followed by immunoblotting with the indicated antibodies. Transfection with the plasmid expressing GFP was used as a negative control. Full-length blots are presented in Supplementary Fig. S8.

Previously we performed a comprehensive screen for human DUBs involved in DSB repair and found that all of four DUBs of the UCH (ubiquitin carboxyl-terminal hydrolase) subfamily consisting of UCHL1, UCHL3, UCHL5 and BAP1, somehow contribute to DSB repair^27^. The function of UCHL1 in DSB repair is still unclear, but it has been established that UCHL5 promotes repair by enhancing DNA-end resection, whereas BAP1 promotes HR via an unknown mechanism^27,28^. Interestingly, a recent report suggested that UCHL3 also plays a role in HR^29^. To further investigate the function of UCHL3 in DSB repair, we sought to identify novel UCHL3 interactors. Mass spectrometry analyses of extracts from cells exogenously expressing green fluorescent protein (GFP)-tagged UCHL3 suggested potential interactions with several DSB repair proteins including Ku70, Ku80 and DNA-PKcs (Fig. 1A and Supplementary Table S1). Subsequently, the interaction between exogenously over-expressed GFP-UCHL3 and endogenous Ku80 and DNA-PKcs was validated by immunoprecipitation-immunoblotting analysis where the interaction occurred even in the presence of ethidium bromide (EtBr), which disrupts non-specific protein-protein interactions mediated by DNA (Fig. 1B and data not shown). These data correlated with the observation that the reciprocal interaction between transiently over-expressed GFP-Ku80 and endogenous UCHL3 was readily detectable (Fig. 1C). The immunoprecipitation of endogenous UCHL3 using an anti-UCHL3 antibody followed by immunoblotting analysis detected the interaction with endogenous Ku80, providing further support that UCHL3 interacts with Ku (Fig. 1D). To examine the nature of this interaction, we constructed various truncated versions of UCHL3 (Supplementary Fig. S1A), which were used as baits for immunoprecipitation experiments. Subsequent immunoblotting analysis with these truncated forms of UCHL3 suggested that the domain comprising the middle part of UCHL3 that contains its catalytic core (residues 75–199) is sufficient to interact with Ku80 (Supplementary Fig. S1B). Further analysis identified two short regions (residues 75–112 and 170–199) that are sufficient for the interaction with Ku80 (Supplementary Fig. S1C). Since these peptides harbour two residues (Cys95 and Asp184) that form the catalytic triad of UCHL3 together with His169 and are thus key for its deubiquitylating activity, we wondered whether the catalytic activity of UCHL3 is required for the interaction with Ku. To investigate this, we mutated Cys95 to Ala as this residue was previously demonstrated to be essential for the enzymatic activity of UCHL3^30^. Interestingly, the UCHL3 C95A mutation abolished its interaction with Ku80 and DNA-PKcs, suggesting that the deubiquitylating activity of UCHL3 is necessary for these interactions (Fig. 1E and data not shown). We also tested whether the ubiquitin-binding ability of UCHL3 was affected in the C95A mutant. Indeed, the catalytically inactive mutant, but not wild-type UCHL3, readily immunoprecipitated mono- and di-ubiquitin moieties, suggesting that persistent ubiquitin binding to the catalytic core of UCHL3 may physically or sterically interfere with the interaction with Ku in cells (Supplementary Fig. S1D). Furthermore, the interaction with Ku80 was constitutive and not affected by IR at least under these conditions, while interactions with downstream c-NHEJ factors XRCC4 and LIG4 were not observed (Fig. 1E). In addition, we used live-cell imaging to examine GFP-UCHL3 recruitment to laser-induced DNA damage sites, a technique commonly used to measure DSB site localization of proteins. While transiently over-expressed wild-type GFP-UCHL3 localized to laser microirradiation sites, recruitment of the catalytically inactive mutant (C95A) was attenuated (Supplementary Fig. S1E, and F), confirming that UCHL3 functions in proximity of DNA lesions and suggesting its recruitment or retention at such DNA breaks rely on its catalytic activity. These data thus suggested that UCHL3 interacts with Ku and/or DNA-PKcs in cells and could have a role at sites of DSBs in a catalytic-activity-dependent manner.

### UCHL3 promotes cell survival after DSB induction and is epistatic with XRCC4

**Figure 2:**
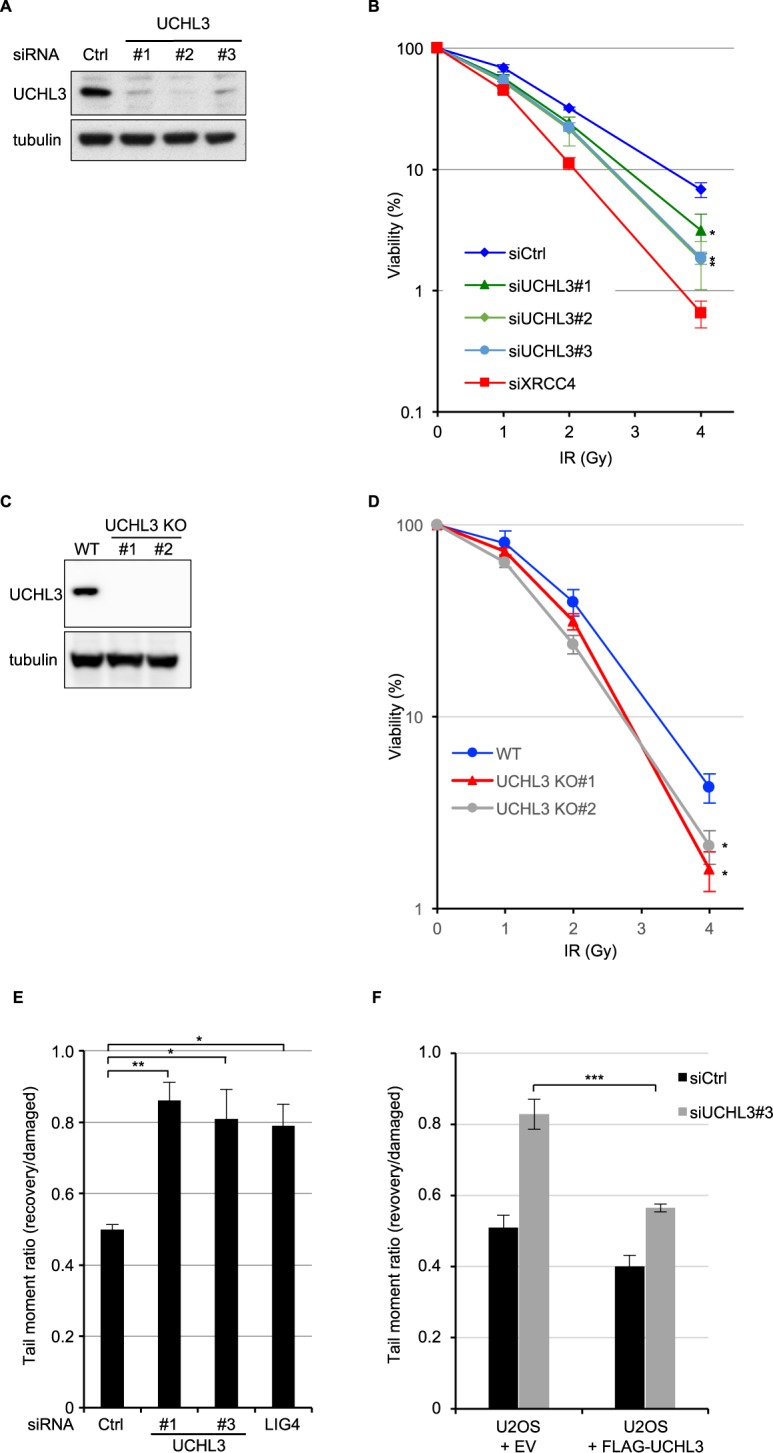
UCHL3 promotes cellular survival after IR and DSB repair. **(A**,**B)** U2OS cells transfected with the indicated siRNAs were processed for immunoblotting **(A)** or were subjected to clonogenic survival assay after IR **(B)** (Mean ± SEM, n = 3). **(C**,**D)** U2OS (wild-type: WT) or U2OS UCHL3 KO (KO#1 and KO#2) cells were processed for immunoblotting **(C)** or were subjected to clonogenic survival assay after IR **(D)** (Mean ± SEM, n = 3). **(E)** U2OS cells transfected with the indicated siRNAs were subjected to neutral comet assay. Efficiency of DSB repair is measured as the tail moment ratio between 2 hours after phleomycin removal and immediately after treatment (Mean ± SEM, n = 3). **(F)** U2OS cells stably expressing FLAG-UCHL3 or FLAG (empty vector: EV) were transfected with the indicated siRNAs and then subjected to neutral comet assay (Mean ± SEM, n = 3). *p < 0.05, **p < 0.01, ***p < 0.001. The tail moments measured immediately after phleomycin treatment, which indicate the amount of generated DSB, are presented in Supplementary Fig. S3. Full-length blots are presented in Supplementary Fig. S9.

**Figure 3:**
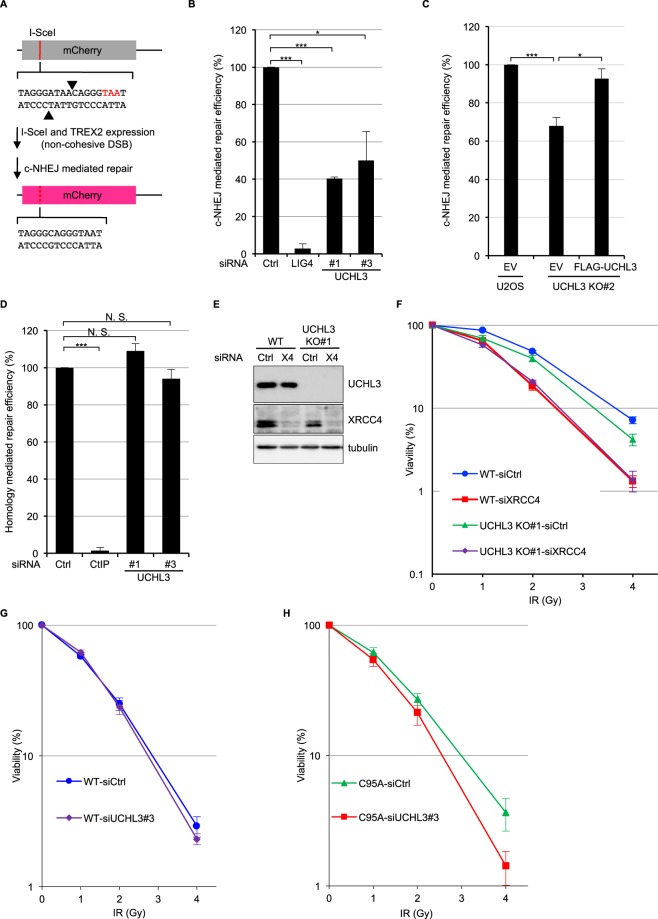
UCHL3 facilitates classical NHEJ. **(A)** Schematic representation of a fluorescent reporter assay measuring c-NHEJ efficiency. I-SceI digestion sites and inserted stop codon are indicated by arrow heads and red characters, respectively. **(B)** U2OS cells transfected with the indicated siRNAs were subjected to c-NHEJ assay. The efficiency of c-NHEJ was normalized to control siRNA-transfected cells and set to 100% (Mean ± SEM, n = 3). **(C)** U2OS or UCHL3 KO#2 cells transfected with the indicated plasmid coding FLAG (empty vector: EV) or FLAG-UCHL3 were subjected to c-NHEJ assay. The efficiency of c-NHEJ was normalized to EV transfected U2OS cells and set to 100% (Mean ± SEM, n = 3). **(D)** U2OS cells transfected with the indicated siRNAs were subjected to direct-repeat GFP assay. The efficiency of homology mediated repair was normalized to control siRNA-transfected cells and set to 100% (Mean ± SEM, n = 3). **(E**,**F)** U2OS (WT) or UCHL3 KO#1 cells transfected with the indicated siRNAs were processed for immunoblotting analysis **(E)** or subjected to clonogenic survival assay after IR **(F)** (Mean ± SEM, n = 3). **(G**,**H)** U2OS cells stably expressing FLAG-UCHL3 (wild-type: WT) **(G)** or catalytically inactive mutant (C95A) **(H)** were transfected with the indicated siRNAs and subjected to clonogenic survival assay after IR (Mean ± SEM, n = 3). *p < 0.05, ***p < 0.001, N. S.; not significant. Full-length blots are presented in Supplementary Fig. S10.

To further explore whether UCHL3 plays a role in cellular responses to DSBs, clonogenic survival assays were performed upon treating cells with various short interfering RNAs (siRNAs) targeting UCHL3. This revealed that UCHL3 depletion moderately sensitised cells to IR (Fig. 2A,B). In line with this, two UCHL3 knockout cell lines (UCHL3 KO#1 and #2), generated by CRISPR-Cas9 mediated gene editing, were more sensitive to IR compared to the parental cell line (Fig. 2C,D). To exclude the possibility that the increased IR sensitivity caused by UCHL3 depletion was due to decreased proliferation or cell cycle issues, cell proliferation and cell cycle profile of these cell lines were examined by 3-(4,5-dimethylthiazol-2-yl)-2,5-diphenyltetrazolium bromide (MTT)-based assay and bromodeoxyuridine (BrdU) incorporation assay, respectively (Supplementary Fig. S2A,B). These data suggested that no significant growth defect was observed with UCHL3 KO#2, while UCHL3 KO#1 proliferated slightly faster than the parental cell line. Consistent with this, a mildly increased S phase population was detected in UCHL3 KO#1, while UCHL3 KO#2 showed no defect. Altogether, these results indicated that the moderately increased IR sensitivity observed upon UCHL3 depletion was likely not due to any growth defects. In light of the above findings, we asked whether UCHL3 directly affected DSB repair by neutral comet assays. Indeed, UCHL3 depletion resulted in significantly reduced DSB repair efficiencies compared to the siRNA control, while comparable levels of DSB were induced in these cells (Fig. 2E and Supplementary Fig. S3A). Furthermore, the observed DSB repair defect upon depleting endogenous UCHL3 using a 3′UTR targeting siRNA (siUCHL3#3) was mitigated by stably expressing FLAG-tagged UCHL3 (Fig. 2F, Supplementary Fig. S3B,C), suggesting that these observations were not due to any siRNA off-target defects. To define which of the two major DSB repair pathways, c-NHEJ or HR, is promoted by UCHL3, we first developed a reporter assay in which simultaneous expression of I-SceI and TREX2 generates a blunt-end DSB and only when the DSB is repaired by classic-NHEJ, is functional mCherry expressed (Fig. 3A). This revealed that depletion of UCHL3 with two independent siRNAs resulted in significantly reduced c-NHEJ efficiencies, while depletion of LIG4 resulted in dramatically reduced c-NHEJ efficiencies, validating that the reporter assay detects LIG4-dependent DSB repair (Fig. 3B). These data were in line with observed c-NHEJ activities being reduced in UCHL3 KO#2 cells, which was largely rescued by exogenously expressed FLAG-UCHL3 (Fig. 3C). On the other hand, HR efficiency assessed by a commonly used direct-repeat GFP assay^31^ was not detectably affected by depleting UCHL3 with various siRNAs, while siRNA-mediated depletion of CtIP, a canonical HR-promoting factor, resulted in significantly reduced HR efficiencies (Fig. 3D). Finally, assessment of whether UCHL3 affected microhomology-mediated end-joining suggested that this alternative DSB repair pathway was not affected by the depletion of UCHL3 (Supplementary Fig. S4). In addition to these findings, we examined whether UCHL3 is epistatic with the c-NHEJ factor XRCC4, which would further confirm a role for UCHL3 in c-NHEJ. Depleting XRCC4 in a UCHL3 knockout cell line (UCHL3 KO#1) did not result in additional sensitisation to IR compared to XRCC4 depletion alone (Fig. 3E,F), implying that UCHL3 functions in the same pathway as XRCC4. Importantly, a stable cell line expressing FLAG-tagged UCHL3 (wild-type), but not enzymatically inactive mutant UCHL3 (C95A), alleviated moderately increased sensitivity caused by depleting endogenous UCHL3 with a 3′UTR targeting siRNA (Fig. 3G,H and Supplementary Fig. S3B). Together, these data support the idea that UCHL3 promotes c-NHEJ, not HR, in a catalytic activity-dependent manner.

### UCHL3 enhances Ku80 chromatin binding and directly deubiquitylates Ku80

**Figure 4:**
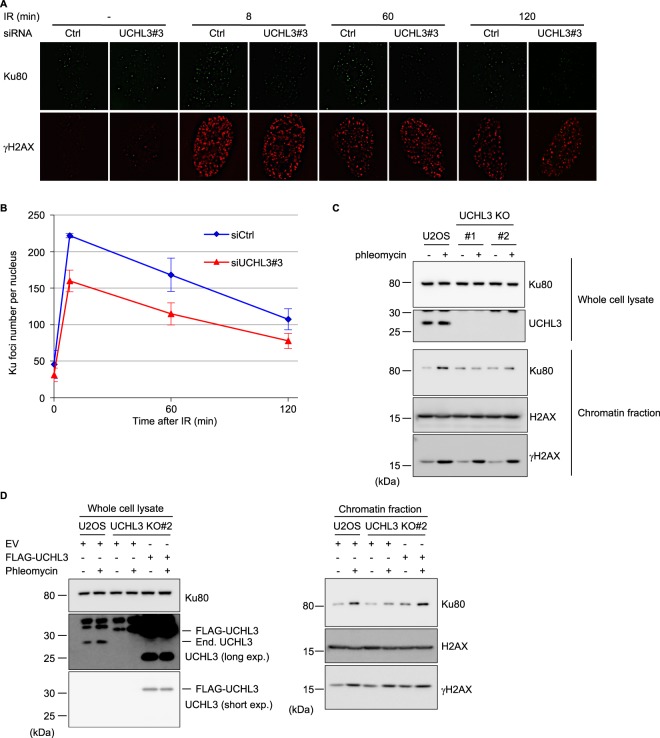
UCHL3 enhances Ku80 chromatin binding upon DSB induction. **(A)** U2OS cells transfected with the indicated siRNAs were exposed to 10 Gy IR and further cultured for various time periods. Immunofluorescent staining with anti-Ku80 antibody and anti-γH2AX antibody was carried out. **(B)** Quantitative analysis of the data shown in **(A)**. The number of Ku80 foci per nucleus was plotted (Mean ± SEM, siCtrl: n = 4, siUCHL3#3: n = 6). **(C)** U2OS cells or UCHL3 KO cell lines (#1 and #2) were subjected to chromatin fractionation assay with phleomycin. Whole cell lysates and chromatin fractions were subjected to immunoblotting with the indicated antibodies. **(D)** U2OS cells or UCHL3 KO cell line (#2) transfected with the indicated plasmids were subjected to chromatin fractionation assay with phleomycin. Whole cell lysates and chromatin fractions were subjected to immunoblotting with the indicated antibodies. For the detection with an anti-UCHL3 antibody, short exposure (short exp.) and long exposure (long exp.) of the film are shown. The exogenously expressed UCHL3 (FLAG-UCHL3) and endogenous UCHL3 (End. UCHL3) are indicated. The immunoblotting with an anti-γH2AX antibody was used as a control for the DNA damage induced.

**Figure 5:**
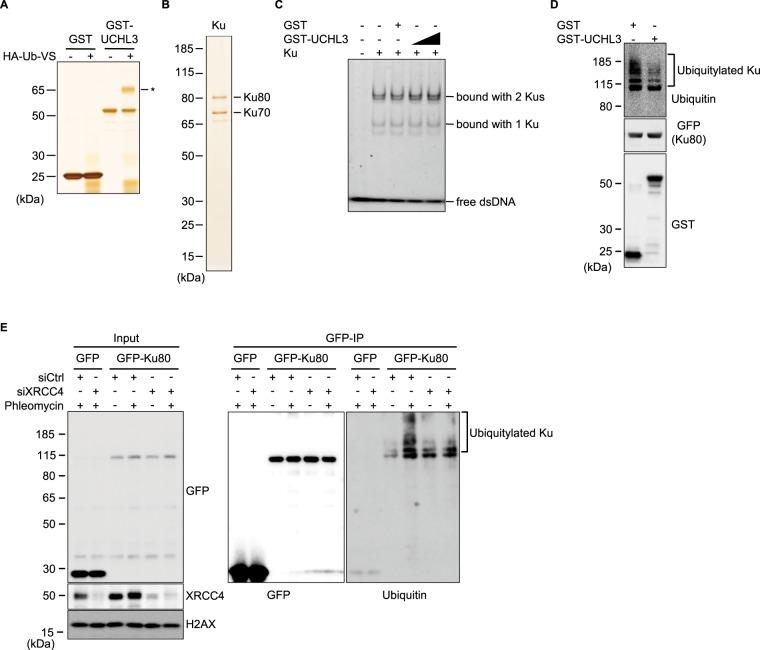
UCHL3 directly deubiquitylates Ku80 ubiquitylation that is dependent on DSB and downstream NHEJ factors. **(A)** Purified GST or GST-UCHL3 was subjected to *in vitro* deubiquitylating enzyme activity assay, verifying purified UCHL3 is enzymatically active. An asterisk indicates HA-Ub-Vs bound UCHL3. **(B)** Silver staining of purified Ku used for electrophoretic mobility shift assay. **(C)** Electrophoretic mobility shift assay was carried out with purified Ku and UCHL3 proteins. The fluorescein labelled double-strand DNA (dsDNA, 50 nM) was incubated with the purified Ku (100 nM) in combination either with GST (600 nM) or GST-UCHL3 (300 or 600 nM). Free dsDNA and dsDNA bound with Ku are indicated. **(D)** GFP-Ku80 was immune-purified from GFP-Ku80 expressing-U2OS cells treated with phleomycin. Purified GFP-Ku80 was subjected to *in vitro* deubiquitylation assay. GFP-Ku80 bound beads were incubated either with GST or GST-UCHL3 and analysed by immunoblotting with the indicated antibodies. The bracket indicates ubiquitylated Ku80. **(E)** U2OS cells stably expressing GFP or GFP-Ku80 were subjected to *in vivo* ubiquitylation assay. Cells were transfected with siRNA targeting XRCC4 or control siRNA and further treated with phleomycin and then subjected to immunoprecipitation (IP) with an anti-GFP antibody. Input and GFP-IPed fractions were subjected to immunoblotting with the indicated antibodies. The bracket indicates ubiquitylated Ku80. Full-length blots are presented in Supplementary Fig. S11.

To examine the effect of UCHL3 on Ku regulation, we investigated IR-induced foci (IRIF) formation of Ku80, which indicates loading of Ku onto DSB sites. UCHL3 siRNA depletion resulted in reduced Ku80 IRIF formation in comparison to control siRNA treatment (Fig. 4A,B). In accordance with reduced Ku80 IRIF, we observed that knockout and knockdown of UCHL3 reduced DSB-induced chromatin binding of Ku80 using chromatin fractionation until 3 hours after phleomycin removal (Fig. 4C and Supplementary Fig. S5). Furthermore, the reduced Ku80 chromatin binding, which was observed in UCHL3 knockout cells, was rescued with the exogenously expressed FLAG-tagged UCHL3 (Fig. 4D). To gain insight into how UCHL3 contributes to Ku80 chromatin binding after DSB induction, firstly, we examined the effect of UCHL3 on the DNA-binding activity of Ku by electrophoretic mobility shift assay with purified these proteins (Fig. 5A–C). This revealed that UCHL3 did not enhance Ku binding to linear dsDNA. Also, UCHL3 itself did not bind to this dsDNA substrate (data not shown). Since it was reported that Ku ubiquitylation is a prerequisite for Ku removal from chromatin, we hypothesized that UCHL3 may enhance Ku80 chromatin binding by antagonizing Ku80 ubiquitylation. To test this hypothesis, we set up an *in vitro* deubiquitylating assay. A cell line stably expressing GFP-Ku80 was incubated with phleomycin to induce Ku ubiquitylation and GFP-Ku80 was purified by immunoprecipitation with an anti-GFP antibody followed by extensive washing with a high salt containing buffer, which allowed us to detect Ku80 ubiquitylation specifically (see Methods, and Fig. 5D,E). Immuno-purified GFP-Ku80 was then incubated with separately purified GST-UCHL3 or GST (Fig. 5D). We observed a reduced ubiquitin signal with GST-UCHL3 but not with GST, suggesting that UCHL3 directly removed ubiquitin from Ku80. Furthermore, we attempted to examine Ku ubiquitylation status in cells upon UCHL3 depletion. However, when we examined Ku80 ubiquitylation with XRCC4 depletion *in vivo*, ubiquitylation of Ku80 was reduced, suggesting that XRCC4-LigaseIV complex recruitment to DSB sites or completion of NHEJ is a prerequisite for Ku ubiquitylation in mammalian cells (Fig. 5E). Therefore, since UCHL3 depletion resulted in reduced Ku80 chromatin binding, it is not possible to assess the effect of UCHL3 on Ku ubiquitylation *in vivo*. In addition to ubiquitylation, NEDDylation, a ubiquitin-like protein modification, was also suggested to play role in Ku ubiquitylation and UCHL3 was previously shown to function as a deNEDDylase^32,33^. We have tested whether UCHL3 affects the NEDDylation status of Cullin 4 A E3 ligase that is involved in Ku ubiquitylation, however, we observed no effect on Cullin 4 A NEDDylation (data not shown). Taken together, these data suggest that UCHL3 could enhance Ku chromatin retention upon DSB induction by deubiquitylating Ku80.

### UCHL3 is phosphorylated in a manner dependent on ATM and its catalytic activity

**Figure 6:**
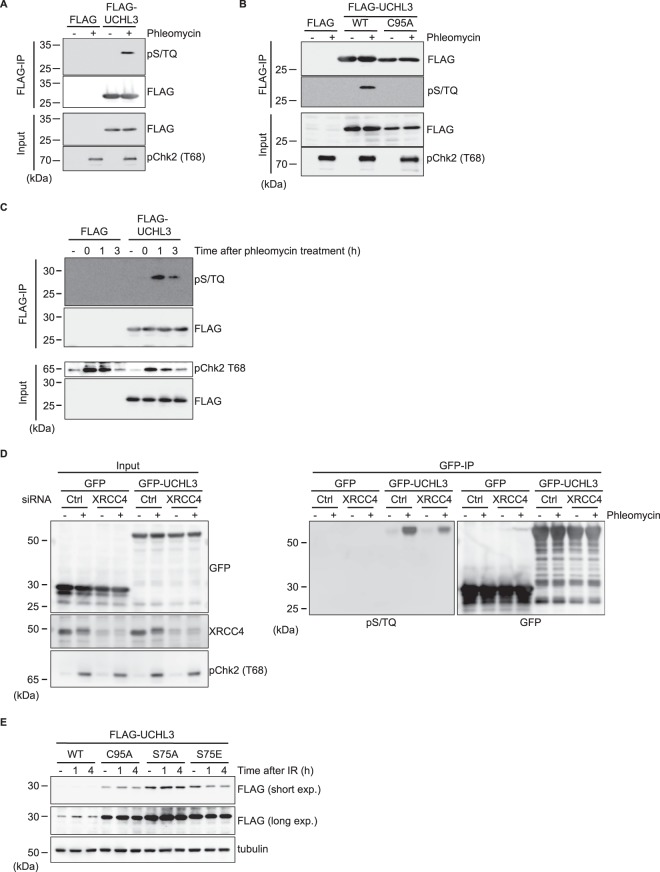
UCHL3 phosphorylation requiring its catalytic activity and downstream NHEJ factors regulates UCHL3 stability. **(A)** U2OS cells transfected with a plasmid expressing FLAG-UCHL3 were treated with phleomycin. Immunoprecipitation with an anti-FLAG antibody was carried out, followed by immunoblotting analysis with the indicated antibodies. Transfection with the plasmid coding FLAG was used as a negative control. **(B)** U2OS cells transfected with a plasmid expressing either FLAG-UCHL3 (wild-type: WT) or catalytically inactive mutant (C95A) were treated with phleomycin or mock treated. Immunoprecipitation with an anti-FLAG antibody was carried out, followed by immunoblotting analysis with the indicated antibodies. Transfection with the plasmid coding FLAG was used as a negative control. **(C)** U2OS cells transfected with a plasmid expressing either FLAG or FLAG-UCHL3 were treated with phleomycin for 1 hour and further cultured for the indicated time periods after removal of phleomycin. Cells were processed for immunoprecipitation with anti-FLAG antibody followed by immunoblotting analyses with the indicated antibodies. **(D)** U2OS cells transfected with the indicated siRNAs were further transfected with a plasmid expressing either GFP-UCHL3 or GFP. Following phleomycin treatment, cell extracts were subjected to immunoprecipitation with an anti-GFP antibody and ensuing immunoblotting analysis with the indicated antibodies. The plasmid expressing GFP was used as a negative control. **(E)** U2OS cells stably expressing FLAG-UCHL3 (WT, C95A, S75A or S75E) were transfected with siRNA targeting endogenous UCHL3. Cells were incubated with cycloheximide (100 μg/ml) for 1 hour prior to IR (10 Gy) and cultured for the indicated time periods after IR. Immunoblotting analyses were performed with the indicated antibodies. For anti-FLAG antibody detection, short exposure (short exp.) and long exposure (long exp.) of films are shown. Full-length blots are presented in Supplementary Fig. S12.

Through ensuing studies, to reveal the molecular mechanism regulating UCHL3 function in the context of c-NHEJ, we speculated that UCHL3 might be post-translationally modified in response to DSB induction. Since UCHL3 was previously listed as a potentially phosphorylated protein in response to IR^34^, and harbours a single SQ site (Ser75), the consensus sequence of ATM, ATR and DNA-PKcs kinases, we tested whether UCHL3 is phosphorylated upon phleomycin treatment. As shown in Fig. 6A, immunoprecipitation-immunoblotting analysis with an anti-phospho S/TQ antibody readily detected FLAG-tagged UCHL3 specifically after phleomycin treatment. The phosphorylation of UCHL3 was also induced by IR treatment (data not shown). Given the findings that UCHL3 interacted with Ku80 and was recruited to sites of damage in a catalytic activity dependent manner (Fig. 1B–E, Supplementary Fig. S1E,F), we investigated whether catalytic activity is required for its phosphorylation. Indeed, DNA damage-induced UCHL3 phosphorylation was not detected with enzymatically inactive UCHL3 (Fig. 6B), suggesting that UCHL3 could be phosphorylated at sites of DNA damage. Interestingly, we found that UCHL3 phosphorylation peaked at 1 hour after phleomycin removal even though the canonical DSB-induced phosphorylation of CHK2 was detected immediately after phleomycin treatment (Fig. 6C). This phenotype prompted us to investigate whether the completion of c-NHEJ or recruitment of downstream factors to DSB sites is prerequisite for UCHL3 phosphorylation. In line with this idea, siRNA mediated depletion of XRCC4 or LIG4, which obstructs NHEJ completion, resulted in attenuated UCHL3 phosphorylation (Fig. 6D and data not shown). These data indicate that phosphorylation of UCHL3 at sites of DNA damage requires downstream c-NHEJ factors. Furthermore, to define which kinase is responsible for UCHL3 phosphorylation, identical experiments were carried out with specific inhibitors targeting ATM or DNA-PKcs kinase activities. Incubating cells with the ATM inhibitor KU-55933 prior to DSB induction almost completely abolished phosphorylation detected with the anti-phospho S/TQ antibody, while the DNA-PKcs inhibitor (NU7441) had no significant effect on this phosphorylation signal (Supplementary Fig. S6A,B). To further establish that the signal detected with the anti-phospho S/TQ antibody indicates phosphorylated UCHL3, immunoblotting was performed with a mutant UCHL3 in which Ser75 was substituted with Ala (S75A). The ensuing results demonstrated that the S75A mutation abolished the signal from the anti-phospho-S/TQ antibody, thus confirming that Ser75 of UCHL3 is phosphorylated in response to DSBs (Supplementary Fig. S6C). We also sought to establish the function of this UCHL3 phosphorylation. In an *in vitro* deubiquitylating activity assay, purified UCHL3 harbouring either S75A (unphosphorylatable mutant) or S75E (phosphomimetic mutant) showed comparable levels of binding to HA-tagged ubiquitin vinyl sulfone (HA-Ub-VS), which covalently binds to DUB active sites, when compared with wild-type UCHL3, suggesting that Ser75 phosphorylation does not affect the intrinsic enzymatic activity of UCHL3 (Supplementary Fig. S7A). Accordingly, the interaction of Ku80 and UCHL3, which requires UCHL3 catalytic activity (Fig. 1E), was not markedly affected when FLAG-tagged phospho-mutants of UCHL3 were examined by immunoprecipitation (Supplementary Fig. S7B). On the other hand, interestingly, we observed that the phosphomimetic mutant of UCHL3 (S75E) became destabilized upon IR treatment, which was not detected with wild-type, catalytically inactive mutant (C95A) nor the unphosphorylatable mutant (S75A) (Fig. 6E). It is worth noting that we have observed that UCHL3 can dimerize *in vivo* (manuscript in preparation). Therefore, we examined the stability of exogenously expressed mutant proteins following depletion of endogenous UCHL3 by siRNA targeting 3′-UTR (Fig. 6E). Altogether, these data suggest that phosphorylation of UCHL3 on S75, which is dependent on ATM, UCHL3′s catalytic activity and downstream NHEJ factors such as XRCC4 and LigaseIV, regulates Ku80 ubiquitylation by regulating UCHL3 stability rather than affecting its deubiquitylating activity after DSB induction.

## Discussion

**Figure 7:**
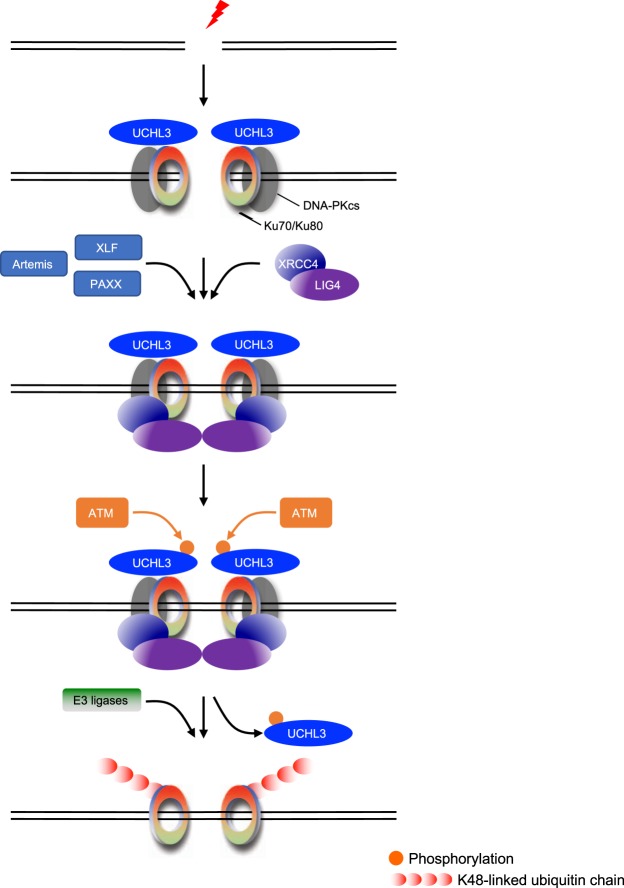
A proposed model for classical NHEJ. UCHL3 could regulate classical NHEJ by antagonizing Ku80 ubiquitylation in its catalytic activity and phosphorylation dependent manner. See main text for detailed description of the proposed model.

Ubiquitylation is a versatile post-translational modification widely used in various cellular signalling including DSB responses. Although Ku ubiquitylation is important for removal of Ku from chromatin at the end of NHEJ, its regulatory mechanism remains unclear with many studies in disagreement on the E3 ligase(s) responsible^20–24,32^. Here we found that UCHL3, a deubiquitylating enzyme, interacts with and can directly deubiquitylate Ku80 in a catalytic activity dependent manner. Furthermore, depletion or knocking out of UCHL3 resulted in reduced Ku80 IRIF and caused moderately increased sensitivity to IR, which was not additive to the cellular sensitivity observed upon XRCC4 depletion. In addition, c-NHEJ efficiency assessed by a fluorescent reporter system was significantly reduced by depleting UCHL3. Our results, therefore, identified UCHL3 as a novel deubiquitylating enzyme counteracting Ku80 ubiquitylation, possibly by cleaving isopeptide- and peptide-linked ubiquitin^35^, and promoting cellular survival after DSB induction via c-NHEJ. Through ensuing studies, we have added mechanistic insights into how UCHL3 regulates Ku80 ubiquitylation. UCHL3 is phosphorylated on S75 in a manner dependent on its catalytic activity, ATM and XRCC4 (as well as LIG4). Although this phosphorylation neither affects the enzymatic activity of UCHL3 *in vitro* nor its interaction with Ku80 in cells, it facilitates destabilization of UCHL3 after DSB induction. Together with our finding that XRCC4 and LIG4 depletion reduced Ku80 ubiquitylation induced by DSB induction (Fig. 5E), collectively these data suggest the following model (Fig. 7). Upon DSB induction, Ku, DNA-PKcs and UCHL3 are recruited to sites of DNA damage. Additional factors such as Artemis, XLF and PAXX are recruited and DSB-ends are processed if necessary. Following the recruitment of XRCC4-LIG4 and ligation of the broken DNA ends, UCHL3 is phosphorylated and destabilized, which tilts the ubiquitylation balance of Ku80 in favour of ubiquitylation by exposing Ku80 to E3 ligases. Finally, ubiquitylated Ku80 is removed from chromatin with the aid of VCP and the proteasome^36^. It is worth noting that even in the presence of a VCP inhibitor, Ku was still removed from chromatin. Therefore, it will be interesting to test whether UCHL3 functions synergistically with VCP. Since NHEJ is the major DSB repair pathway and is completed within relatively short time periods (approximately 30 minutes) in mammalian cells, topologically trapped Ku that can interfere with transcription and DNA replication also needs to be removed within this time period. Our data supports the idea that Ku80 ubiquitylation is induced by loss of UCHL3 triggered by ATM-mediated phosphorylation, and E3 ligases targeting Ku80 might be constitutively activated. This scenario would be plausible for achieving rapid extraction of Ku from chromatin to avoid the deleterious effect of topologically trapped Ku after completion of NHEJ. Such dissociation of DUBs induced by ATM-dependent phosphorylation has been reported for another UCH family DUB, BAP1, the substrate of which is ubiquitylated H2A. Phosphorylation of BAP1 that promotes HR resulted in reduced chromatin binding of BAP1^28^. This may suggest that some ubiquitylations are regulated by release of the DUB from its substrate in the context of a DSB response. In human cells, depletion of E3 ligases responsible for Ku ubiquitylation (at least RNF8 and RNF126) resulted in delayed DSB repair^20,21^. It is also suggested that NHEJ completion is not required for Ku ubiquitylation in the *Xenopus laevis* egg extract system^24^. These findings may suggest that Ku ubiquitylation is important not only for removing Ku from chromatin, but also for properly positioning downstream factors for the ligating of two broken DNA ends. However, whether Ku ubiquitylation regulates recruitment or translocation of XRCC4-LIG4 or completion of NHEJ in mammalian cells remains elusive^37^. We also found that DSB-induced Ku ubiquitylation requires XRCC4-LIG4, suggesting that the molecular mechanism of Ku ubiquitylation might be different between mammalian cells and *Xenopus laevis* egg extract system. In addition, while our results provided molecular insight into how Ku is removed from chromatin via Ku ubiquitylation, this raises an important question: how is the completion of NHEJ sensed in the nucleus? To address this and other questions, further research will be required. Since various E3 ligases have been suggested for Ku ubiquitylation, it would be intriguing to investigate whether UCHL3 targets the ubiquitylation of Ku by specific E3 ligase(s). In addition, since the increased sensitivity to IR caused by UCHL3 depletion was moderate and the human genome encodes around 100 DUBs, it would be interesting to examine if any other DUBs can deubiquitylate Ku. Furthermore, UCHL3 was recently suggested to facilitate HR by deubiquitylating RAD51 in a DSB-dependent manner via phosphorylation on the same site that we identified (S75)^29^, however, we did not observe any defects in HR caused by UCHL3 depletion, nor did we detect any interaction with RAD51 in our mass spectrometry analyses. It is unclear how our respective studies could come to such different conclusions at this point, so further replication studies will be required in future. Furthermore, it has been suggested that DNA-PKcs also plays roles in responses to stalled replication forks besides DSB repair^38,39^. Our mass spectrometry and ensuing immunoprecipitation analysis indicated that UCHL3 could interact with DNA-PKcs, possibly suggesting context specific functions of UCHL3 for maintaining genome integrity. Although it will be intriguing to reveal how UCHL3 may contribute to independent DSB repair pathways, our findings in this study highlight the importance of UCHL3 for maintaining genome integrity by regulating the major DSB repair pathway, c-NHEJ.

## Methods

### Cell lines and cell culture

All cell lines were cultured at 37 °C in a 5% CO_2_ humidified atmosphere. U2OS cell lines stably expressing GFP, GFP-Ku80, RFP-53BP1 or FLAG-UCHL3 (WT, C95A, S75A and S75E) were cultured in Dulbecco’s modified Eagle medium (DMEM, Nacalai Tesque) containing 10% fetal bovine serum (FBS, Sigma-Aldrich), 100 U/ml penicillin (Nacalai Tesque), 100 μg/ml streptomycin (Nacalai Tesque), 584 μg/ml L-glutamine and 500 μg/ml Geneticin (Thermo Fisher Scientific). U2OS stably expressing the HR reporter direct repeat-GFP, EJ2-GFP reporter^40^ and U2OS UCHL3 knock out cell lines were cultured in identical media with 1 μg/ml puromycin (InvivoGen) instead of Geneticin.

### Mass spectrometry and data analysis

Cell extracts were prepared using a Benzonase-based method (see below) from U2OS cells transiently transfected with pEGFP-C1-UCHL3 or pEGFP-C1 as a negative control. Immunoprecipitates with anti-GFP antibody were washed six times with IP lysis buffer [20 mM Tris-HCl (pH 7.5), 2 mM MgCl_2_, 0.5% NP-40, 10% glycerol] containing 300 mM NaCl, 1 x protease inhibitor cocktail, ethylenediaminetetraacetic acid (EDTA)-free (PI, Roche), 10 mM NaF (Nacalai Tesque), 20 mM N-ethylmaleimide (NEM, Nacalai Tesque) and 0.25 mM phenylmethylsulfonyl fluoride (PMSF, Sigma-Aldrich), and then collected by boiling at 95 °C for 10 min with 1 x Laemmli SDS buffer [62.5 mM Tris-HCl (pH 6.8), 2% sodium dodecyl sulfate, 10% glycerol and 0.02% bromophenol blue, 6.25% β-mercaptoethanol and 300 mM NaCl]. IP-enriched eluate material was subjected to two rounds of chloroform-methanol precipitation^41^. Precipitated protein material was digested in-solution and analyzed using nano-liquid chromatography tandem mass spectrometry (nano–LC-MS/MS) as described previously^42^.

### Cell extract preparation and immunoblotting analysis

Except for sample preparation for mass spectrometry analysis, *in vivo* ubiquitylation assays, *in vitro* deubiquitylation assays and chromatin fractionation assays, cell extracts were prepared with CSK buffer [10 mM PIPES (pH 6.8), 3 mM MgCl_2_, 1 mM ethylene glycol tetraacetic acid (EGTA), 0.1% Triton X-100 and 300 mM sucrose] containing 300 mM NaCl, 1 x PI, 10 mM NaF, 20 mM NEM and 0.25 mM PMSF. Cells were washed twice with ice-cold PBS and incubated with an appropriate volume of CSK buffer for 1 hour on ice with occasional mixing. Soluble fractions were collected by centrifugation at 20,000 x g for 10 min at 4 °C. For mass spectrometry analysis, *in vivo* ubiquitylation assays and *in vitro* deubiquitylation assays, cells were washed twice with ice-cold PBS and collected with an appropriate volume of ice-cold PBS followed by centrifugation at 10,000 × g, 1 min. Cells were lysed with IP lysis buffer containing 40 mM NaCl, 1 × PI, 10 mM NaF, 20 mM NEM, 0.25 mM PMSF and 50 U/ml Benzonase (Merck Millipore) and incubated at room temperature for 5 min. Soluble fractions were prepared by rotating at 4 °C for 1 hour after adjusting NaCl concentrations to 300 mM followed by centrifugation at 20,000 × g for 10 min at 4 °C. For chromatin fractionation, cells were washed twice with ice-cold PBS and collected with an appropriate volume of ice-cold PBS followed by centrifugation at 10,000 × g, 1 min. Cells were then pre-extracted with CSK buffer containing 100 mM NaCl, 1 × PI, 10 mM NaF, 20 mM NEM, 0.25 mM PMSF and 0.3 mg/ml RNaseA for 5 min at room temperature. Chromatin fractions (pellet fraction) were prepared by centrifugation at 20,000 × g, 10 min at 4 °C and then pre-extracted once more. Residual chromatin fractions were washed once with identical buffer without RNaseA and solubilized by sonication (TOMY, UD-100, 40% output, 30 sec). Protein concentration of cell extracts was determined with Coomassie Protein Assay Reagent (Thermo Scientific) with bovine serum albumin standard (Takara). The antibodies used in immunoblotting are described in Supplementary Table 2.

### Immunoprecipitation

Soluble fractions from cell extracts prepared with CSK buffer, were immunoprecipitated with an anti-GFP antibody coupled to magnetic beads (GFP-Trap_MA, ChromoTek) or anti-FLAG antibody coupled to agarose beads (anti-FLAG M2 Affinity Agarose Gel, Sigma-Aldrich) by rotating overnight at 4 °C. For the immunoprecipitation of endogenous UCHL3, an anti-UCHL3 antibody (Proteintech, 12384-1-AP) and Protein G Sepharose beads (GE Healthcare) were used. The beads were washed six times with CSK buffer containing 300 mM NaCl and bound proteins were eluted by boiling at 95 °C for 10 min with 1 x Laemmli SDS buffer for GFP-Trap_MA or incubating with 500 μg/ml FLAG peptide (Sigma-Aldrich) for 30 min on ice for samples where anti-FLAG antibody was used for immunoprecipitation. For the detection of ubiquitylated proteins in *in vivo* ubiquitylation assays, beads were washed twice with IP lysis buffer containing 300 mM NaCl, three times with same buffer containing 1 M NaCl and once with the original buffer, followed by elution as described above.

### Transfection of Plasmids and siRNAs

Cells were transfected with the plasmids or siRNAs (40 nM at final concentration) by Mirus TransIT-LT1 (Mirus Bio LLC) or HiperFect (Qiagen) according to the manufacturer’s instruction, respectively. See Supplementary Table 3 for the siRNAs used in this study.

### Preparation of recombinant proteins

For recombinant glutathione S-transferase (GST) tagged UCHL3 (wild-type, C88A, S75A and S75E) expression, *E*. *coli* cells [BL21(DE3)] was transfected with pGEX6P-1 coding for the above mentioned UCHL3s. Cells carrying pGEX6P-1-UCHL3s were grown in 1 liter of Super Broth medium [5 g NaCl, 32 g Bacto-trypton and 20 g yeast extract per liter (pH 7.0)] at 25 °C until OD_600_ reached 0.6 and further culture for 6 hours in the presence of 100 μM isopropylthioglucoside (IPTG). The cell pellet was washed with ice-cold 10% glycerol, suspended in 1.5 M NaCl containing lysis buffer [50 mM Tris-HCl (pH 7.5), 0.5 mM EDTA, 5 mM CaCl_2_, 2 mM dithiothreitol (DTT), 0.5 mM PMSF, 10% glycerol, and 1 x PI]. The soluble fraction was obtained by sonication followed by centrifugation at 20,000 x g for 30 min and loaded onto an Econo-pack column (Bio-Rad) filled with Glutathione Sepharose 4B (GE Healthcare) that was equilibrated with lysis buffer. The column was successively washed with lysis buffer containing 1.5 M, 0.3 M and 0.15 M NaCl. Bound proteins were eluted by incubating with elution buffer [50 mM Tris-HCl (pH 8.0), 10% glycerol, 0.15 M NaCl, 2 mM DTT, 0.5 mM PMSF and 20 mM reduced glutathione] for 10 min at room temperature. This elution step was repeated twice, and a second fraction of elution was used for *in vitro* assays. Recombinant Ku was purified as described elsewhere (J. L. Wang *et al*., 2018, Nat. Struct. Mol. Biol., in press). The purity of the eluted fraction was examined by SDS-PAGE followed by silver staining with 2D-SILVER STAIN-II according to manufacturer’s instructions (Cosmo Bio). Protein concentration of the eluted protein was determined by measuring the absorbance at 280 nm with NanoPhotometer (IMPLEN).

### *In vitro* deubiquitylating enzyme activity assay

HA-tagged ubiquitin vinyl sulfone (2 μM, Enzo Life Sciences) was incubated with 400 ng of purified either GST or GST-UCHL3 in 30 μl of reaction buffer [50 mM Tris-HCl (pH 7.5), 50 mM NaCl and 1 mM DTT]. Following the incubation for 2 hours at 37 °C, the reaction was terminated by adding 10 μl of 4 × Laemmli SDS buffer and boiling at 95 °C for 5 min. The mixture was analyzed by SDS-PAGE followed by silver staining.

### Live cell imaging

U2OS cells stably expressing RFP-53BP1 in 35 mm glass-bottom dishes (WillCo-dish) were transfected with 1 μg of expression plasmids coding for GFP-UCHL3 (wild-type) or (C95A mutant) with Mirus TransIT-LT1 and further cultured for 48 hours in the presence of 10 μM 5-Bromo-2′-deoxyuridine (BrdU). On the day of analysis, the media was replaced with phenol red-free DMEM (Sigma-Aldrich) supplemented with 10% FBS, penicillin, streptomycin and 25 mM HEPES buffer (pH 7.0–7.6, Sigma-Aldrich). DNA damage was induced by irradiating cells through a UPlanSApo 60x/1.35 oil objective lens with UV-A laser (405 nm) using a IX81 confocal microscope (Olympus) equipped with a 37 °C heating stage (Ibidi). The laser output was set at 400 mW with 50 scans of 10 msec/pixel. Prior to DNA damage induction and 15 min after damage induction, images were taken and analyzed by using FluoView 1000 software (Olympus). The signal intensity was quantified by using ImageJ.

### Clonogenic cell survival assay

Clonogenic viability was examined using a colony formation assay. Briefly, forty-eight hours after the initial transfection with siRNAs, cells were seeded in 6 well plates and treated with acute IR on the following day. For the assay with knock out cell lines, cells were seeded in 6 well plates one day before irradiation. Colonies were stained with crystal violet solution [2% crystal violet (Sigma-Aldrich) in 10% ethanol] 10–13 days after IR treatment.

### Establishment of UCHL3 knock out cell line

The gRNA sequence (5′-TCCGAGTTACTCATGAGACC-3′) was cloned between the BamHI site and the BsmBI site of pCas-Guide vector (Origine). Homology arm sequences flanking the PAM site were cloned into HR410PA-1 (System Biosciences). U2OS cells co-transfected with these plasmids were cultured for 14 days with puromycin and screened for loss of UCHL3 expression by immunoblotting.

### Neutral comet assay

Seventy-two hours after 40 nM siRNA transfection, the cells were incubated with 40 μg/ml phleomycin for 2 hours or mock incubated. Following phleomycin treatment, cells were washed twice with PBS and cultured for an additional 2 hours. The cells were subsequently washed twice with PBS (−) and collected by trypsinization. Approximately 5 × 10^3^ cells in 10 ml of PBS (−) were mixed with 90 μl of LMAgarose (Trevigen), placed on GelBond Film (Lonza), covered with a 22 mm cover slide (VWR International) and left at 4 °C for 1 hour. Upon removal of the cover slide, the cells were lysed with Lysis Solution (Trevigen) at 4 °C for 1 hour. Following a wash with TBE [90 mM Tris-Borate (pH 8.3) and 2 mM EDTA], the samples were subjected to electrophoresis at 35 V, for 7 min in TBE. After washing with TBE, samples were fixed with 70% ethanol for 5 min at room temperature and dried overnight. The nuclei were stained with SYBR Green I (Invitrogen) in 10 mM Tris-HCl (pH 7.5) and 1 mM EDTA for 5 min at 4 °C. Images were taken with a fluorescent microscope IX71 (Olympus) with Cell^F software (Olympus). Tail moments were measured by using CometScore software (TriTek). The means of tail moment of at least 50 cells were measured. Efficiency of DSB repair was determined as the tail moment ratio between 2 hours after phleomycin removal and immediately after treatment.

### Classical NHEJ reporter assay

Classical NHEJ efficiency was investigated by the transfection of pmCherry-C1 harbouring the insertion of an I-SceI recognition site, which introduced a stop codon at the 5′ terminus of the mCherry gene, together with transient expression of the I-SceI restriction enzyme and TREX2 3′ to 5′ exonuclease. For U2OS cells, the initial siRNA transfection was carried out 24 hours before the second siRNA transfection followed by plasmid transfection with further 6 hours later. Forty-eight hours after plasmid transfection, the ratio of mCherry-expressing cells was determined by Flow cytometer (Beckon Dickinson).

### Ku IRIF

Ku IRIF were examined as described previously^43^. Briefly, cells grown on coverslips were irradiated with 10 Gy of IR or mock irradiated. Cells were washed twice with PBS and then extracted with preextraction buffer [10 mM PIPES (pH 7.0), 100 mM NaCl, 300 mM sucrose, 0.7% Triton X-100, 0.3 mg/ml RNase A and 3 mM MgCl_2_]. Following washing with PBS, cells were fixed with 2% paraformaldehyde for 15 min at room temperature and further permeabilized with 0.2% Triton X-100 for 5 min. Hereafter, cells were washed with PBS containing 0.1% Tween 20 between each procedure. After blocking with PBS containing 5% BSA and 0.1% Tween 20 (blocking solution), cells were incubated with primary antibodies and then Alexa Fluor 488 or 594 fluorophores (Thermo Fisher Scientific) in blocking solution. Nuclei were stained with 0.5 μg/ml 4′,6-diamidino-2-phenylindole for 10 min at room temperature and mounted with Vectashield (Vector Laboratories). Images were taken on the Deltavision OMX V3 and analyzed by Volocity software (PerkinElmer). At least 40 nuclei were counted for each experiment.

### Electrophoretic mobility shift assay

A blunt-ended 50 bp DNA fragment with fluorescein at the 5′-end was prepared by annealing fluorescein labelled top strand and unlabeled bottom strand. A binding reaction was performed for 30 min on ice in a 20 μl solution containing 20 mM Tris-HCl (pH 7.5), 50 mM KCl, 0.1 mM DTT, 5% glycerol, 10 μg/ml BSA, fluorescein labelled DNA (50 nM), Ku (100 nM) and GST-UCHL3 (300 or 600 nM). GST (600 nM) was used as a negative control. The resulting reaction mixtures were subjected to 5% native PAGE at 50 V for 180 min at 4 °C with 0.5 x TBE buffer [49.5 mM Tris, 49.5 mM boric acid and 1 mM EDTA]. Immediately after electrophoresis, images were taken with LAS3000 (GE Healthcare).

### *In vitro* deubiquitylation assay

Stable cell lines expressing GFP-Ku80 from four 100 mm dishes were treated with 500 μM phleomycin for 1 hour. Immediately after phleomycin treatment, extracts were prepared and subjected to immunoprecipitation with anti-GFP antibody conjugated beads (see cell extract preparation and immunoprecipitation sections). After a washing step, immunopurified proteins were equilibrated with deubiquitylation buffer [50 mM Tris-HCl (pH 7.5), 150 mM NaCl, 3 mM EDTA, 0.5% NP-40 and 1 mM DTT] by further washing three times and suspended in the identical buffer. Equally separated beads were incubated with 1.53 μg of GST or GST-UCHL3 at 30 °C for 20 min in 41 μl reaction scale and then the reaction was terminated by adding 13.7 μl of 4 x Laemmli SDS buffer followed by boiling at 95 °C for 10 min. The resulting mixtures were analyzed by immunoblotting.

### *In vivo* ubiquitylation assay

Stable cell lines expressing GFP or GFP-Ku80 were transfected with indicated siRNAs seventy-two hours prior to phleomycin treatment (500 μM, 1 hour) or mock treatment. Extracts were subjected to immunoprecipitation with anti-GFP antibody (see above) and analyzed by immunoblotting.

### Cell growth assay and cell cycle analysis

Cells were cultured in 96 well plate and subjected to cell growth assay with Cell Counting kit-8 according to the manufacturer’s instruction (Nacalai Tesque). Cell cycle analysis with BrdU incorporation was carried out as previously described^27^.

### Antibodies and siRNAs

Antibodies and siRNAs used in this research are listed in Supplementary Table S2 and Supplementary Table S3, respectively.

### Statistical analyses

All statistical analyses were carried out by two-sided Student’s t-test.

## Electronic supplementary material

Supplementary information

## Data Availability

All data and materials are available upon request.
