# Supplementary material for: The deubiquitylating enzyme UCHL3 regulates Ku80 retention at sites of DNA damage
Source: Sci Rep. 2018 Dec 17;8:17891. doi: 10.1038/s41598-018-36235-0 (PMC6297141; doi:10.1038/s41598-018-36235-0)
Supplement: Supplementary file 1 — Supplementary information [file 41598_2018_36235_MOESM1_ESM.pdf]

Supplementary information

The deubiquitylating enzyme UCHL3 regulates Ku80 retention at sites of DNA damage

Ryotaro Nishi, Paul W. G. Wijnhoven, Yusuke Kimura, Misaki Matsui, Rebecca Konietzny, Qian Wu, Keisuke Nakamura, Tom L. Blundell, and Benedikt M. Kessler

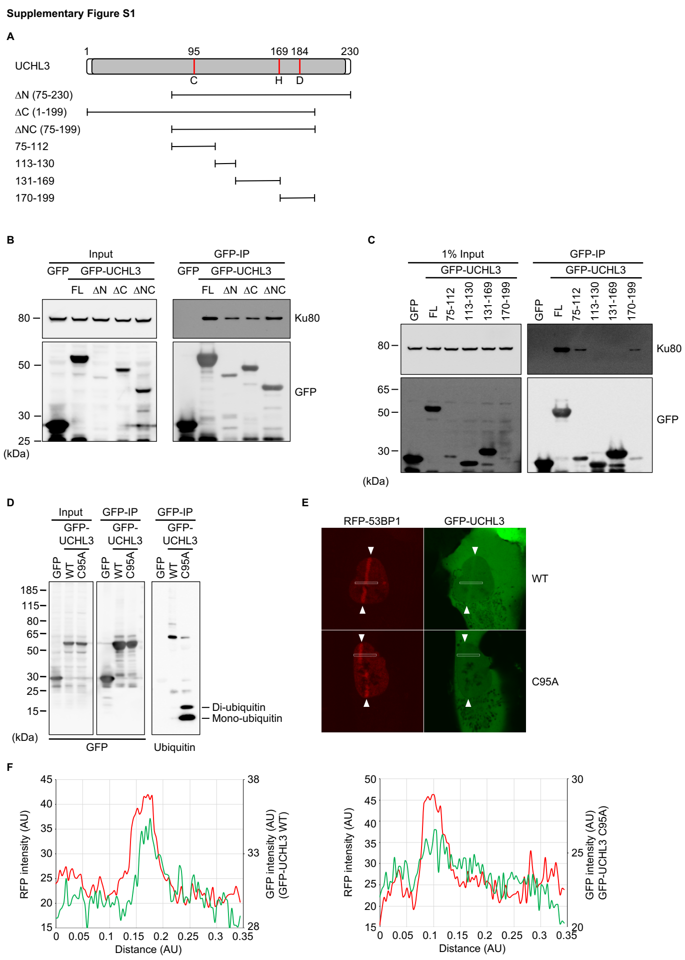

Supplementary Figure S1. Catalytic activity of UCHL3 is required for the interaction with Ku80

(A) Schematic representation of deletion mutants of UCHL3. **(B-D)** U2OS cells were transfected with the plasmids expressing various GFP-UCHL3 constructs as indicated. Immunoprecipitation with an anti-GFP antibody was carried out, followed by immunoblotting analysis with the indicated antibodies. The plasmid expressing GFP was used as a negative control. **(E)** U2OS cells stably expressing RFP-53BP1 were transfected with a plasmid coding GFP-UCHL3 (WT) or catalytically inactive mutant (C95A). Cells were subjected to live cell imaging and images were taken 15 min after laser micro irradiation. Arrow heads indicate laser micro irradiation sites. White boxes indicate area used for the quantification of signal intensity. **(F)** The signal intensity of GFP or RFP was plotted against distance. Left and righ panel show the results for GFP-UCHL3 (WT) and GFP-UCHL3 (C95A), respectively.

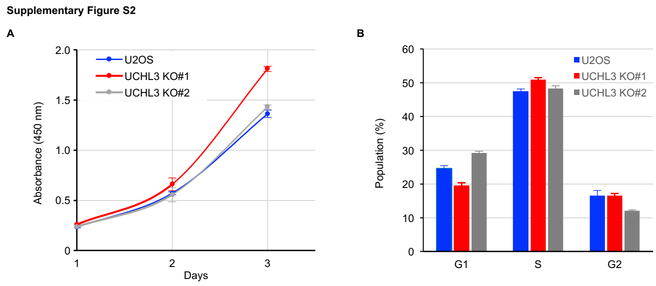

Supplementary Figure S2. UCHL3 KO cell lines did not show growth defect

The indicated cell lines were subjected to cell growth assay **(A)** or cell cycle analysis **(B)** (Mean ± SEM, n=3). **(A)** Absorbance at 450 nm on the indicated day was plotted. **(B)** Cellular population of the indicated cell cycle phase was plotted.

Supplementary Figure S3

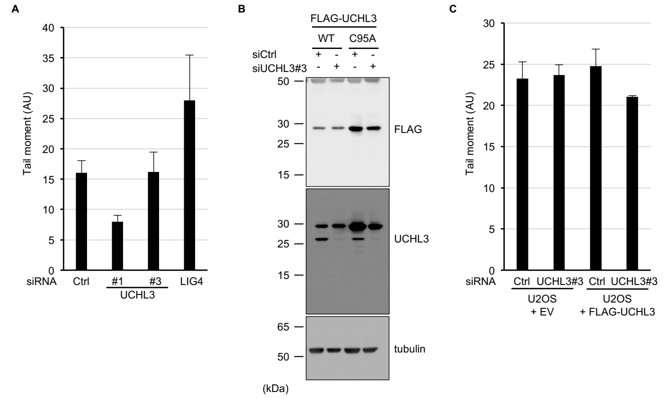

Supplementary Figure S3. Controls for neutral comet assay

(A) U2OS cells transfected with the indicated siRNAs were subjected to neutral comet assay and tail moments measured immediately after phleomyciin treatment, which indicate the amount of generated DSB, were plotted (Mean ± SEM, n=3). **(B)** U2OS cells stably expressing FLAG-UCHL3 (wild-type: WT) or catalytically inactive mutant (C95A) were subjected to immunoblotting analysis with the indicated antibodies. **(C)** U2OS cells stably expressing FLAG (empty vector: EV) or FLAG-UCHL3, which were transfected with the indicated siRNAs, were subjected to neutral comet assay and tail moments measured immediately after phleomyciin treatment were plotted (Mean ± SEM, n=3). **(A)** and **(C**) are corresponding controls to Figure 2E and 2F, respectively.

Supplementary Figure S4

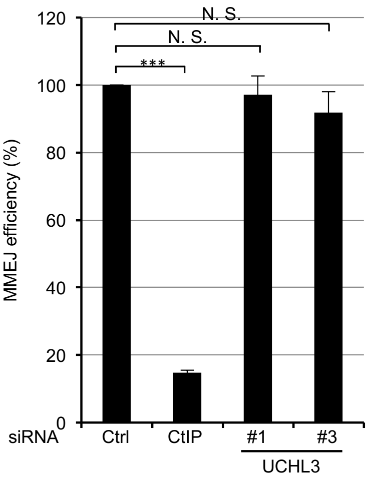

Supplementary Figure S4. Efficiency of microhomology-mediated end joining was not affected by UCHL3 depletion

EJ2-GFP reporter cells transfected with the indicated siRNAs were subjected to microhomology-mediated end joining (MMEJ) assay. The efficiency of MMEJ was normalized to control siRNA-transfected cells and set to 100% (Mean ± SEM, n=3). *** p< 0.001, N. S.; not significant.

Supplementary Figure S5

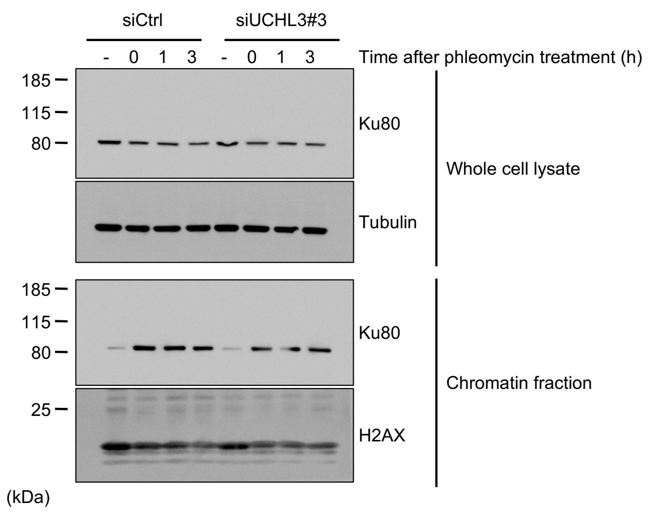

Supplementary Figure S5. UCHL3 enhances Ku80 retention on chromatin upon DSB induction

U2OS cells transfected with the indicated siRNAs were subjected to chromatin fractionation assay with phleomycin. Whole cell lysates and chromatin fractions were subjected to immunoblotting with the indicated antibodies.

Supplementary Figure S6

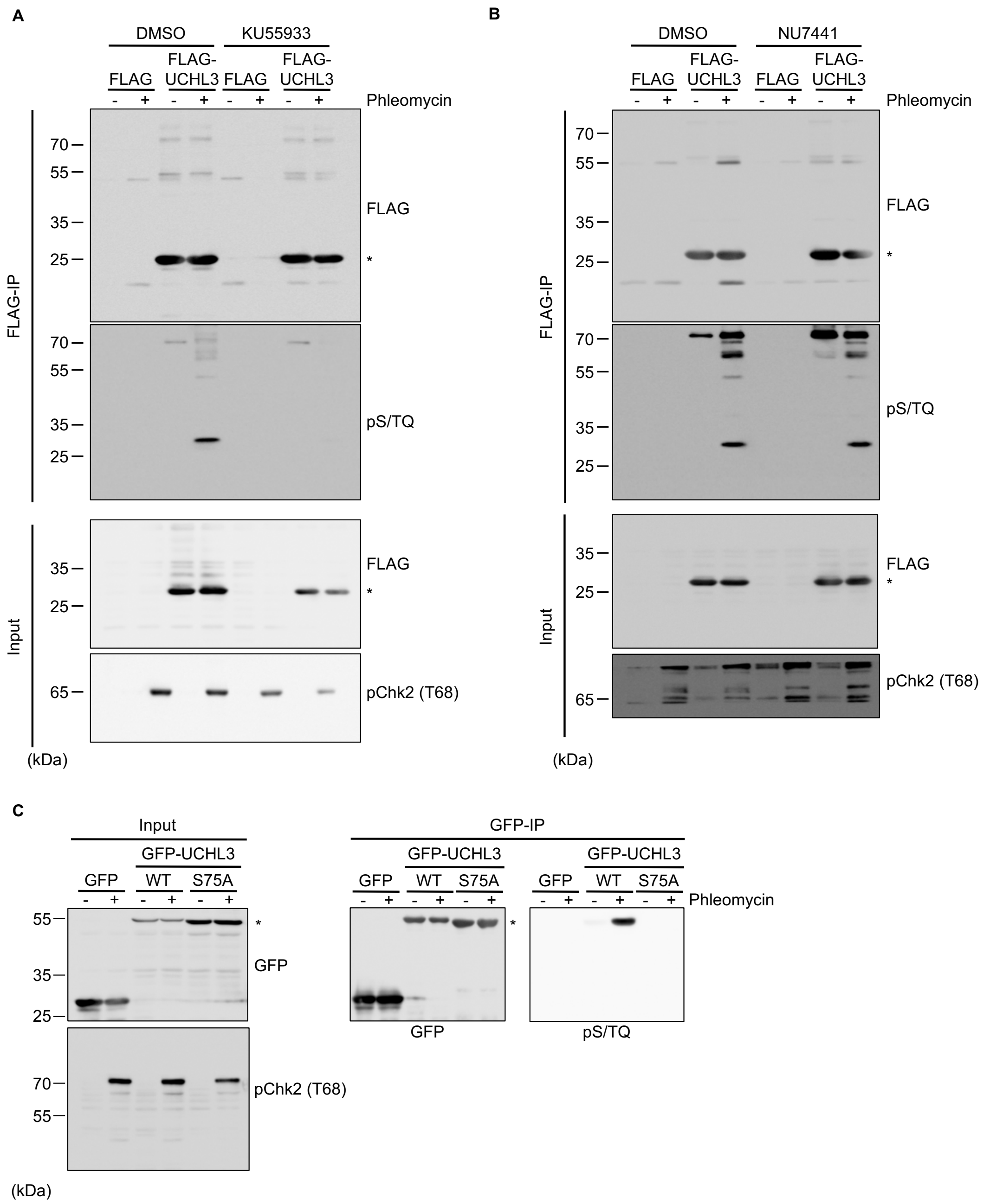

Supplementary Figure S6. UCHL3 phosphorylation on S75 is mediated by ATM kinase

(A, B) U2OS cells transfected with a plasmid expressing FLAG-UCHL3 were treated with DMSO, ATM inhibitor (KU55933) **(A)**, or DNA-PKcs inhibitor (NU7441) **(B)** prior to incubation with phleomycin. Immunoprecipitation with an anti-FLAG antibody was carried out, followed by immunoblotting analysis with the indicated antibodies. Transfection with the plasmid coding FLAG was used as a negative control. **(C)** U2OS cells transfected with the plasmid expressing GFP-UCHL3 (wild-type: WT) or S75A mutant (S75A) were treated with

phleomycin. Immunoprecipitation with an anti-GFP antibody was carried out, followed by immunoblotting analysis with the indicated antibodies. The plasmid expressing GFP was used as a negative control. The asterisks indicate FLAG or GFP-tagged UCHL3.

Supplementary Figure S7

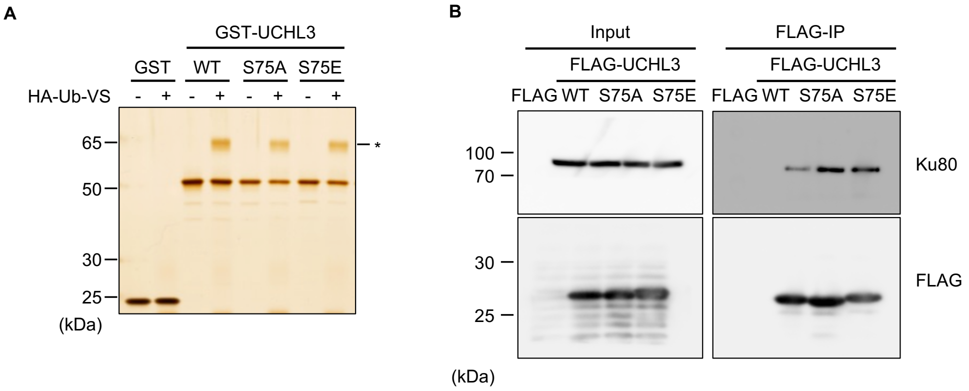

Supplementary Figure S7. Phosphorylation of UCHL3 does not affect its catalytic activity nor its physical interaction with Ku80

(A) Purified GST or GST-UCHL3 (WT, S75A or S75E) was subjected to *in vitro* deubiquitylating enzyme activity assay. HA-Ub-VS bound UCHL3 is indicated by an asterisk. **(B)** U2OS cells transfected with a plasmid expressing FLAG-UCHL3 (WT, S75A or S75E) were subjected to immunoprecipitation with anti-FLAG antibody followed by immunoblotting analysis with the indicated antibodies. The plasmid expressing FLAG was used as a negative control.

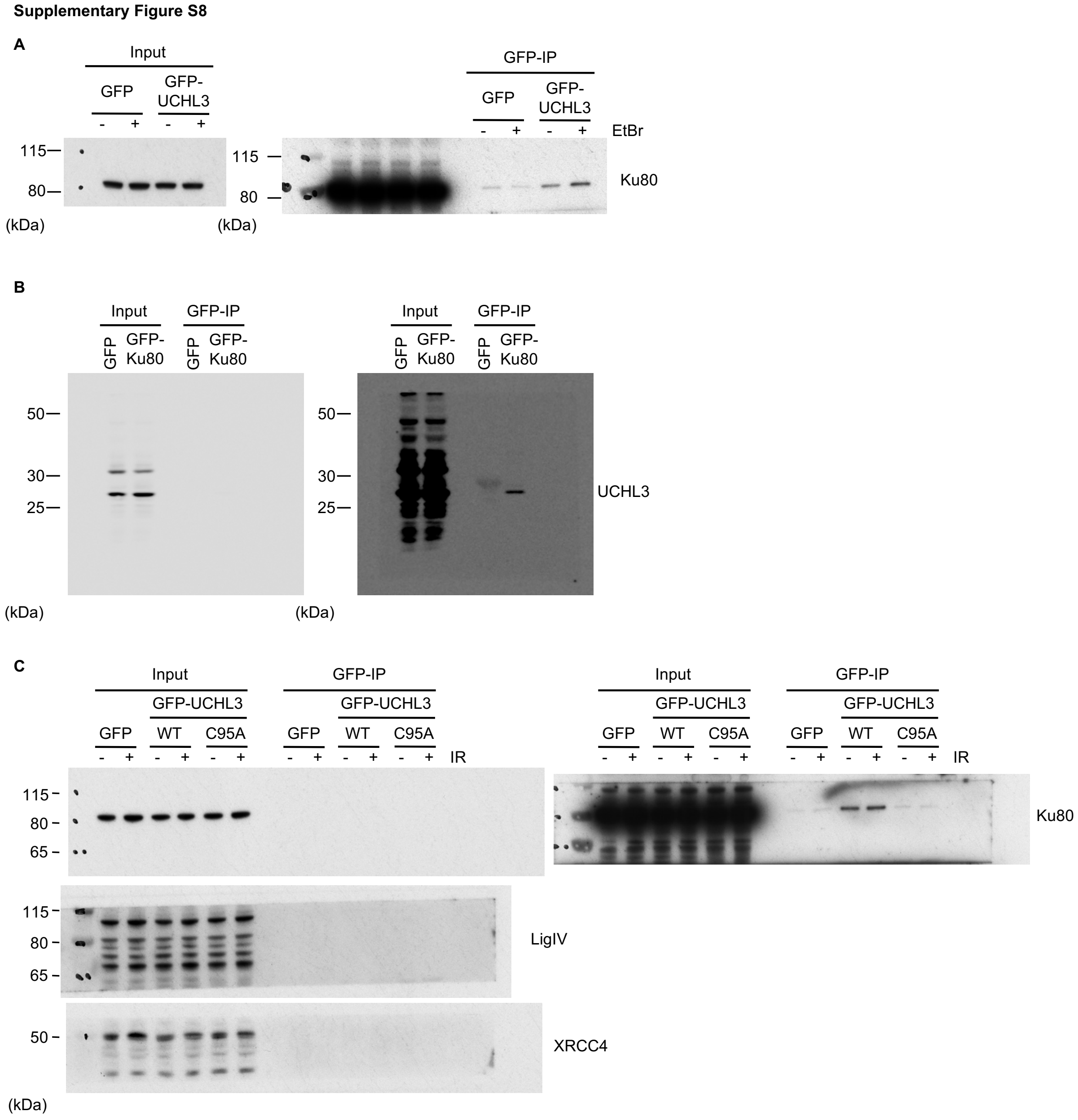

Supplementary Figure S8. Uncropped images for Figure 1.

Uncropped gels for Figure 1B **(A)**, 1C **(B)** and 1E **(C)** are shown.

Supplementary Figure S9

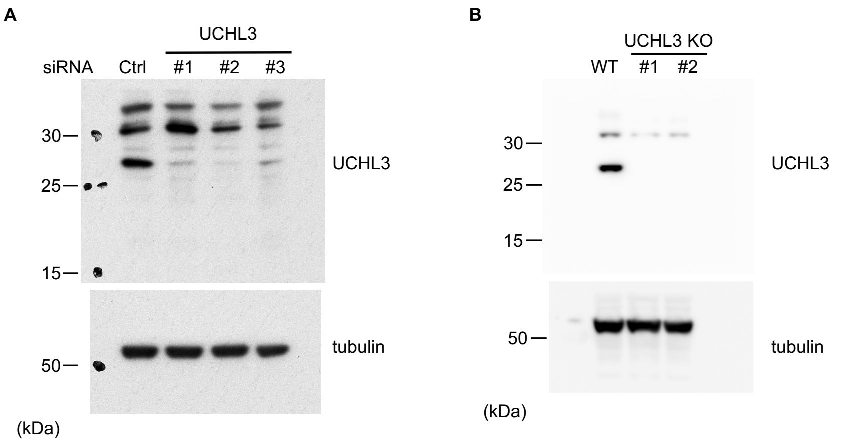

Supplementary Figure S9. Uncropped images for Figure 2.

Uncropped gels for Figure 2A **(A)** and 2C **(B)** are shown.

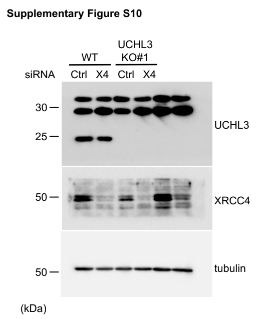

Supplementary Figure S10. Uncropped images for Figure 3.

Uncropped gels for Figure 3E are shown.

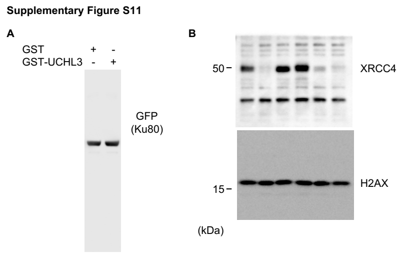

Supplementary Figure S11. Uncropped images for Figure 5.

Uncropped gels for Figure 5D **(A)** and 5E **(B)** are shown.

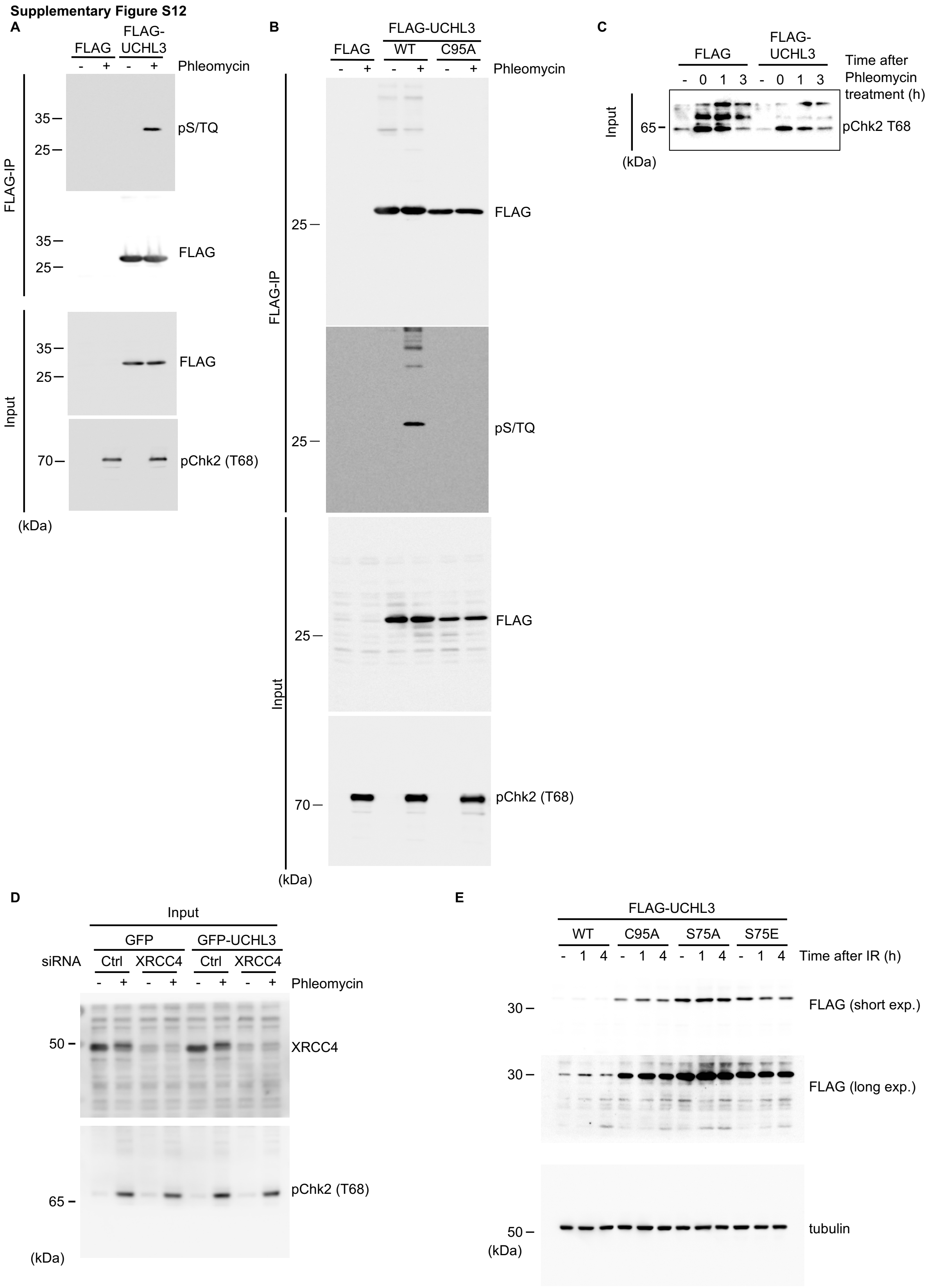

Supplementary Figure 12. Uncropped images for Figure 6.

Uncropped gels for Figure 6A **(A)**, 6B **(B)**, 6C **(C)**, 6D **(D)** and 6E **(E)** are shown.

Accession numbers	Gene names	Numbers of matches
P15374	*UCHL3*	314
P63261	*ACTG1*	88
P62736	*ACTA2*	66
P78527	*PRKDC*	47
P68371	*TUBB4B*	45
P11142	*HSPA8*	40
Q00610	*CLTC*	36
P49327	*FASN*	33
P08107	*HSPA1A*	32
Q9NR30	*DDX21*	31
Q9BUF5	*TUBB6*	28
P04843	*RPN1*	28
P55084	*HADHB*	27
P22570	*FDXR*	26
Q13748	*TUBA3C*	24
P50991	*CCT4*	24
P49368	*CCT3*	23
Q14103	*HNRNPD*	23
P02538	*KRT6A*	22
Q9UHB6	*LIMA1*	22
P13010	*XRCC5*	22
P68366	*TUBA4A*	21
P14625	*HSP90B1*	21
P14866	*HNRNPL*	21
P12236	*SLC25A6*	21
P00558	*PGK1*	19
P60842	*EIF4A1*	18
P05783	*KRT18*	18
P34897	*SHMT2*	18
P17844	*DDX5*	18
P12956	*XRCC6*	17
Q15233	*NONO*	17
Q99877	*HIST1H2BN*	16
Q9H0U4	*RAB1B*	16
P20700	*LMNB1*	16
P07900	*HSP90AA1*	16
P55795	*HNRNPH2*	16
P11387	*TOP1*	16
Q9BWM7	*SFXN3*	15
P51114	*FXR1*	15
P26641	*EEF1G*	15
Q99832	*CCT7*	15
Q12906	*ILF3*	15
Q12905	*ILF2*	14
P30837	*ALDH1B1*	14
P62244	*RPS15A*	14
P31040	*SDHA*	14
O95831	*AIFM1*	14
P32322	*PYCR1*	14
P40926	*MDH2*	13
P54886	*ALDH18A1*	13
P40227	*CCT6A*	13
Q9UJS0	*SLC25A13*	13
O00571	*DDX3X*	13
P51149	*RAB7A*	13
O00567	*NOP56*	13
O95573	*ACSL3*	13
P42765	*ACAA2*	13
O00116	*AGPS*	13
Q9Y2J2	*EPB41L3*	13
Q9Y5M8	*SRPRB*	12
P38919	*EIF4A3*	12
P14868	*DARS*	12
P26373	*RPL13*	12
P62906	*RPL10A*	12
Q08945	*SSRP1*	12
P06733	*ENO1*	12
Q13838	*DDX39B*	11
O00148	*DDX39A*	11
P62136	*PPP1CA*	11

Supplementary Table S1. List of UCHL3 interactors

The list of proteins obtained from a mass spectrometry analysis of immunoprecipitate with an anti-GFP antibody from GFP-UCHL3 expressing cells, but not detected with GFP-expressing cells. The proteins detected with more than 10 peptide matches are shown with accession numbers, numbers of matches and gene names.

Antibody target	Supplier	Catalog Number	Clone number	Application	dilution
Ku80	Thermo Scientific	MS-285-P0	111	IB, IF	2,000
DNA-PKcs	Thermo Scientific	MS-369-P0	18-2	IB	1,000
Ligase IV	Riballo et al., 1999			IB	3,000
XRCC4	Abcam	ab145		IB	1,000
H2AX	Abcam	ab11175		IB	5,000
GFP	Roche	11814460001	7.1 and 13.1	IB	1,000
UCHL3	Cell Signaling Technology	3525		IB	500
UCHL3	Proteintech	12384-1-AP		IP	
FLAG	Sigma-Aldrich	F3165		IB	2,000
α-tubulin	Sigma-Aldrich	T9026	DM1A	IB	5,000
ubiquitin	Cell Signaling Technology	3933		IB	1,000
GST	Nacalai	04435-84	GS019	IB	1,000
Phospho-ATM/ATR substrate Motif	Cell Signaling Technology	6966		IB	1,000
phospho-Chk2 (t68)	Cell Signaling Technology	2661		IB	1,000

Supplementary Table S2. List of antibodies used in this research

Immunoblotting: IB. Immunofluorescent staining: IF. Immunoprecipitation: IP. Antibody dilutions are also shown for the relevant application(s).

siRNA	Sequence (5’ to 3’)	Supplier
Ctrl	AACGUACGCGGAAUACUUCGA	Eurofins
UCHL3#1	CAGGGACAAGAUGUUACAUCA	Eurofins
UCHL3#2	UAGAAGUUUGCAAGAAGUUUA	Eurofins
UCHL3#3	CUGCCAUACACUAACUCAA	Eurofins
Ligase IV	AGGAAGUAUUCUCAGGAAUUA	Eurofins
XRCC4	AUAUGUUGGUGAACUGAGA	Eurofins

Supplementary Table S3. List of siRNAs used in this research
